# Unsupervised feature extraction of aerial images for clustering and understanding hazardous road segments

**DOI:** 10.1038/s41598-023-38100-1

**Published:** 2023-07-05

**Authors:** John Francis, Jonathan Bright, Saba Esnaashari, Youmna Hashem, Deborah Morgan, Vincent J. Straub

**Affiliations:** 1 The Alan Turing Institute, London, NW1 2DB UK; 2 Accountable, Responsible and Transparent AI CDT, Department of Computer Science, University of Bath, Bath, UK

**Keywords:** Civil engineering, Imaging and sensing

## Abstract

Aerial image data are becoming more widely available, and analysis techniques based on supervised learning are advancing their use in a wide variety of remote sensing contexts. However, supervised learning requires training datasets which are not always available or easy to construct with aerial imagery. In this respect, unsupervised machine learning techniques present important advantages. This work presents a novel pipeline to demonstrate how available aerial imagery can be used to better the provision of services related to the built environment, using the case study of road traffic collisions (RTCs) across three cities in the UK. In this paper, we show how aerial imagery can be leveraged to extract latent features of the built environment from the purely visual representation of top-down images. With these latent image features in hand to represent the urban structure, this work then demonstrates how hazardous road segments can be clustered to provide a data-augmented aid for road safety experts to enhance their nuanced understanding of how and where different types of RTCs occur.

## Introduction

The field of remote sensing, which is concerned with detecting and monitoring the physical characteristics of an area from a distance, has grown substantially with the proliferation of freely accessible high-quality aerial imagery. Remote sensing as a domain within computer vision has recently led to advances in agriculture^1^, climate change^2^, transportation^3^, and disaster response^4^. With governments such as the UK aiming to harness the power of machine learning (ML) to deliver “first-class public services”^5^, there is a need for academic research that demonstrates how available data and technologies within remote sensing can help allow for the improved provision of public services. This project takes on the important safety issue of road traffic collisions (RTCs) as a case study for exploring how old challenges can be tackled from fresh perspectives using aerial imagery alongside ML methodologies.

Globally, approximately 1.3 million people die each year because of RTCs, and a majority of these deaths fall among vulnerable road users such as pedestrians and cyclists^6^. In the UK, the country we focus on here as a case study due to the government's prioritisation of road safety and availability of aerial imagery, fatal or serious injuries occur on public roads every 16 min^7^. In 2020, the United Nations General Assembly resolved to halve the number of global deaths and injuries from RTCs by 2030, noting that the “overwhelming majority” of these cases are preventable^8^. To achieve this ambitious goal, new technologies and data will be required to enhance road safety experts’ implementation of RTC interventions.

The study of RTCs has a long history with research dating back to the early days of motorisation in the first two decades of the twentieth century. While early research focused on the characteristics that make up “accident-prone drivers”, today’s road safety research is more focused on how to best implement policies and interventions aimed at creating a safe road system that works for a variety of road users^9^. Although ML has been utilized in recent research concerned with predicting future RTCs or identifying hazardous road locations^10,11^, this project shows how aerial imagery can equip public officials, who already have some knowledge of which points within the road network are dangerous, with a new perspective. Specifically, we demonstrate how to leverage ML methods to cluster hazardous road segments solely on their built form which can help improve qualitative analysis of and intervention planning for reducing future RTCs. To do this, we utilize unsupervised machine learning techniques to extract latent features from aerial images and then leverage hierarchical cluster techniques to group hazardous road segments. We show how aerial image data can provide nuance to understanding how and where different types of RTCs occur, which can in turn allow for improved targeting of interventions designed at tackling this classical issue.

Aerial image data presents unique challenges in terms of extracting data and meaning. On the one hand, aerial images offer incredible, high-resolution data on the built environment. However, extracting meaning from image data is not as straightforward as working with prototypical tabular data. Image data requires linkages to existing knowledge (road networks in our case) as well as novel metrics of similarity in order to enable comparisons between images. To address these challenges, we present a pipeline that extracts meaning from aerial images with minimal supervision, a process which has been shown to have widespread utility^12^, and focus on three case study locations within the UK, Cambridge, Gloucester, and Oxford, to demonstrate the application of the pipeline for road safety analysis.

The rest of this paper is structured as follows. We first review related work on the built environment and road safety, especially prior research that has sought to use aerial imagery and ML methods. Then, we describe the data and methods used in our paper. To apply our methods, we present the results of our analytic pipeline for the three cities we use as case studies: Cambridge, Gloucester, and Oxford. Finally, we discuss the results and conclude by outlining the limitations of our study and considering future directions for similar methodologies in other public settings.

### Literature review

Prior research on the built environment has found success by conceptualizing features at the macro- or neighbourhood-level and at the micro- or street-level^13^. At the neighbourhood-level, features such as urban density and street connectivity have been found to be related to outcomes such as walkability^14^. At the street-level, features such as street trees and street width have been associated with housing price^15^ and air quality^16^, respectively. This study extracts both macro and micro features of the built environment from aerial images to give a more holistic understanding of the larger geographic context around hazardous road segments.

Studies of the built environment have previously made efforts to quantify the many physical attributes of the urban environment. Access to imagery when analysing the built environment has been lauded for better enabling standardised assessments, in turn facilitating comparability across other image-based studies of the built-environment^17,18^. Ewing and Handy^19^, for instance, found mixed success using human annotators to measure more than 100 characteristics of the built environment from video clips. Other researchers have used machine learning to try and computationally measure some of these features from street view images^20^. Measuring features such as building facades, street condition, or even temporally dynamic features, such as the number of parked cars, is tricky, with various cultural or selection biases introduced no matter the method used for quantification. By relying solely on the latent visual features residing within images, this study attempts to bypass some of the issues that arise from manually deriving measures of the built environment.

Given the potential of ML to address global challenges^12^, using ML methods alongside imagery to study the built environment has become popular in recent years to study crime incidents^21^, urban morphology^22^, as well as human perceptions of their urban environments^23^. In many ways, raw imagery provides a more unfiltered view of the built environment as opposed to the more common annotated datasets such as land use or street features in a tabular format.

One popular area of study that relies heavily on data about the built environment is road safety research. In their comprehensive review of RTC research, Gutierrez-Osorio & Pedraza^24^ found that governmental data such as road infrastructure design features were among the most commonly used sources. Importantly, numerous features of the built environment have been found to contribute to RTCs. Looking at RTCs involving pedestrians, Guo et al.^25^ found that more densely connected road networks were related to higher incidence rates. Meanwhile, a qualitative study of fatal collisions in the UK found that excessive speed on road bends was a clear factor in many fatal accidents, particularly in low-lit areas during hours of darkness and on more rural roads^26^.

More recently, deep learning methods have become common in road safety research, primarily as a means for making predictions with tabular data to better capture the spatial and temporal aspects of RTC data. Convolution neural networks^10^, negative binomial models^27^, and long short-term memory networks^28^ have all been shown to predict RTCs with relatively high accuracy and claim to be more effective than classical regression techniques. Other methods such as decision trees^29^ and extreme gradient boosting^30^ have been used to explore the relative importance of various built environment attributes on RTC frequency and injury severity respectively. Closely related to this project, prior research by Zhang et al.^11^ used graph neural networks to extract latent visual features from satellite imagery in order to identify hazardous traffic locations in conjunction with social media data. Their work showed that ML-derived image features out-performed conventional and deep learning models for traffic risk forecasting in New York. Additionally, the authors observed that “locations with similar accident rates tend to share similar visual features” (p. 2)^11^. While Zhang et al.^11^ focused on mining historical data to make predictions about future hazardous locations, our work hopes to help officials better understand past RTCs to allow for improved interventions that can prevent future RTCs.

An area of major concern for road safety research is omitted variable bias. Often, many factors affect the likelihood of a RTC which do not have detailed data available for analysts. Statistical methods that are meant to account for this unobserved heterogeneity often struggle with increased complexity, complicating interpretability and transferability^31^. While it is extremely difficult and costly to collect data on some of these unknown factors, such as the mental state of drivers, raw aerial image features allow for a more complete view of aspects of the built environment that contribute to each incident.

One less studied area is the use of unsupervised ML methods to extract latent image features from the built environment. In one study, Singleton et al. measure local spatial structures from the latent image features derived from Sentinel 2 satellite data, creating a new measure of geodemographic classification^32^. Another study extracted the latent features from street view imagery to allow for a more interpretable method of predicting street quality and street network attributes^33^. Further work described the areas around leisure and retail amenities using latent image features extracted from storefront images^34^. One common thread among these works is the use of dimension reduction techniques such as k-means or principal component analysis (PCA) to allow for an easier interpretation of the latent image features.

Clustering algorithms are among the most used analytic tools among RTC researchers^24^. Clustering algorithms calculate the similarity across features using a distance function to partition objects into clusters. Unlike supervised ML tasks such as classification, clustering does not require data points to have a predefined target category to train the algorithm on. One limitation of commonly used clustering algorithms is the possibility that there can be a multitude of valid possible solutions. Additionally, just because clusters can be formed, does not mean that the clusters themselves are meaningful. This study utilizes agglomerative clustering with Euclidean linkage distance, which is a bottom-up hierarchical clustering technique that tends to form higher quality clusters although it can be expensive computationally^35^. Another benefit of agglomerative clustering is that the method is computationally robust, so the same result is achieved every time.

Taken together, prior work has shown that combining aerial imagery with ML has the potential to help address global challenges relating to the built environment, specifically, RTCs. While urban planners have long studied the physical characteristics that influence traffic patterns and RTCs, harnessing aerial imagery and ML can enable governments to make more accurate forecasts and devise policies informed by city-level data. Importantly, rolling out such technologies also creates new dilemmas, especially if the focus is on ML solely as a predictive tool. For example, what should a city planner do if presented with a statistical probability of 60%—or even 97%—that a road segment will be the site of a future RTC? Should the government invest more resources in interventions to make that location safer for pedestrians, or divert them away? Here, we do not undertake such a normative stance or offer a predictive analysis, instead, we seek to show the potential of using ML to help road safety experts understand their cities better by deriving useful insights about the built environment from aerial imagery and historical RTC data. As such, our results demonstrate the potential of unsupervised ML techniques in extracting aerial image features that can effectively cluster hazardous road segments.

## Study sites and data

In this section, we will describe the study sites and associated data used in our paper. Data for each of the study sites are taken from three sources: aerial imagery from the EDINA Aerial Digimap Service; street network data from OpenStreetMap; and RTC data from the UK Department for Transport (DfT). These three data sources are described in turn below.

### Study sites

Three mid-sized UK towns were selected for this analysis as they represent the average urban environment in the UK. We selected three study sites to bolster the number of possible training images for our feature-extraction models, while also allowing us to demonstrate that the pipeline can be utilized in locations with varying local priorities. To ensure extraction of the most robust features, we wanted to select locales with similarly structured built environments. Cambridge, Gloucester, and Oxford were chosen as they are comparable in terms of population and land area. This, coupled with their proximity to one another and shared morphological heritage^36^ allows us to reasonably assume that their built environments will contain similar types of features. Table 1 provides some additional contextual transport information for each of the study sites.

**Table 1:** Study Site Details. Population and Land Area^40^; Local authority highways and transport expenditure 10-year average 2009- 2018^41^; Road Condition Statistics 2017–2021^42^; Estimated yearly traffic calculated using data and methods described in^43^; Average Road Speed derived from OpenStreetMap.

		Cambridge	Gloucester	Oxford
Population		144,714	132,538	160,021
Land Area		41 km^2^	41 km^2^	46 km^2^
Transport Expenditures (thousands of £)		15,400	11,400	20,700
Road Condition (5-year avg.)	% Major Roads needing maintenance	3	2	4.2
	% Minor Roads needing maintenance	6.4	5	6.6
	% Local Roads needing maintenance	19	12.4	21.4
2021 Estimated Yearly Traffic (millions of km)	Bicycles	22.4	1.6	16.7
	Motorcycles	3.4	3.9	4.1
	All Motor Vehicles	556.7	737.4	550.8
Average Road Speed (km/h)		45.8	53.9	41.7

While these three study sites share similar structural components, the deployment of these components differ due to varying local conditions and priorities. In Cambridge, the latest local plan emphasized a revised design approach to improve safety by creating a ‘low-speed environment’^37^. As part of this, Cambridge is instituting policies which hope to build on the culture of cycling and walking within the city, noting their belief that roads designed to have a speed limit of 20 mph can generally accommodate cyclists without additional provisions^37^. In Gloucester, road safety policies are aimed at generating a 50% reduction in road fatalities by 2032. Gloucester is building a “safety culture” when it comes to their roads, with targeted education programmes utilized as one of their top strategies^38^. In Oxford, improving junction safety is a key area of focus as junction collisions have been rising over the past few years. A key component of the Oxford plan is to create systems which can better understand the causes of collisions, noting that their most serious accidents tend to involve pedestrians and cyclists^39^.

### Aerial image data

Vertical aerial image RGB data for each of the study sites in this project were retrieved from the EDINA Aerial Digimap Service at 25 cm resolution^44^. In total 540 true orthorectified 1 km by 1 km images were acquired that have been processed to retain their geometric fidelity. The aerial imagery were captured to an absolute accuracy of 1.1 m root mean square error and contain less than 5% snow and cloud cover per image. Image data varies in availability from year to year, the full details of which can be found in Supplementary Table S1. Multiple years of images were acquired to bolster the number of available training samples, however for final analyses only a single year’s worth of images from each locale are included.

### Street network data

Street network data for this project was retrieved from OpenStreetMap^45^ for a single time point in June 2020. We observed minimal change in these street networks over the time period of the acquired aerial image data, so a central time point was selected. Only roads which are accessible to cars were included in this analysis, so the network was filtered to remove cycleways, footpaths, and other types of non-vehicular roads.

### Road traffic collision data

RTC data is based on the point-level incidents provided by the DfT road accidents and safety statistics publication for the years 2017–2020 and compiled with the stats19 R package which enables access to the UK’s official RTC database^46^. Importantly, the RTC data used in this article only includes collisions involving personal injury on public roads which have been reported to police within 30 days. In the UK, data on vehicular damage-only RTCs do not generate a police report and incident counts are not published. While this paper focuses only on RTCs involving personal injury, we believe these incidents are the most critical to mitigate and have the potential to benefit most from this analysis. Besides the location of the RTC, descriptive variables are also recorded for each incident including the type of road user involved in the incident. Full details on the RTCs included can be found in Supplementary Table S2.

## Methodology

**Figure 1:**
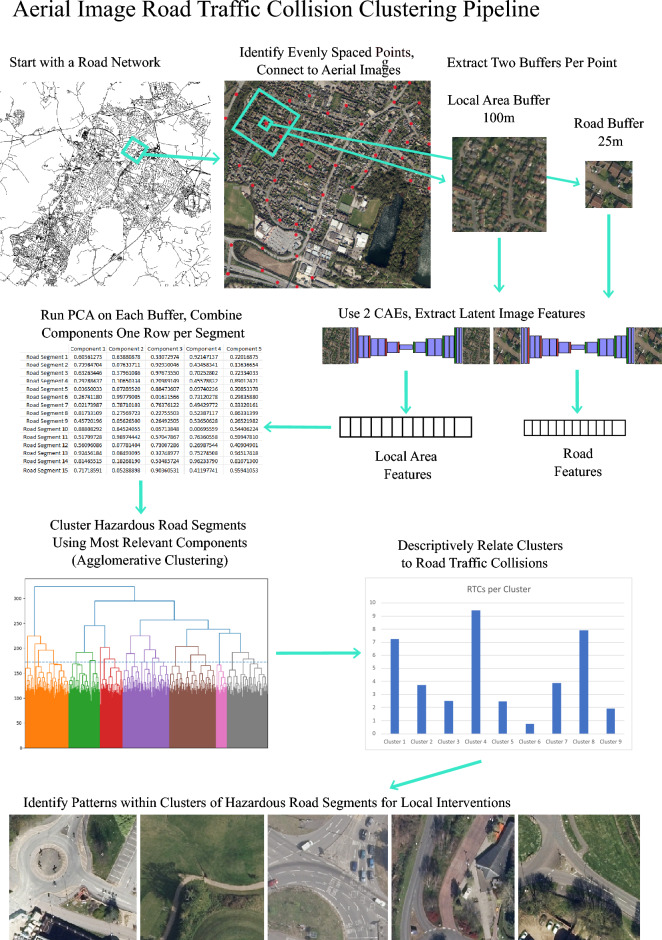
Aerial Image Road Traffic Collision Methodological Pipeline. Plots and images shown here were generated for illustrative purposes only. Maps were generated in R using software v4.3.0 (https://www.R-project.org/) and imagery were sourced from the EDINA Aerial Digimap Service (https://digimap.edina.ac.uk/aerial).

Figure 1 shows the methodological pipeline employed for this analysis. First, road networks and aerial images were combined into one dataset of evenly spaced road segment points, which were further linked to RTC data. Then, a convolutional autoencoder (CAE), PCA, and hierarchical clustering are used to extract image features from the data before grouping road segments for analysis and interpretation. We describe each of these steps in turn in the sections below.

### Data combination

Starting with the street network data, points were sampled evenly along the street network every 50 m. Two square buffers, one with a radius of 25 m and one with a radius of 100 m, were created for each point. We expect that having two buffers will allow for better feature extraction overall, as the 25-m buffer should capture micro features about the road itself, while the 100-m buffer should capture macro features about the local street network and the urban form. The small buffer size was chosen to capture the full width of the dataset’s widest road sample, while the large buffer size was chosen as it has been used previously as a search radius for traffic accident research^47^. Each of the square buffers was matched to an aerial image and clipped to create two sets of image patches for feature extraction. The 100 m buffers were then resampled from a 25 cm resolution to a one metre resolution so both sets of images would have a size of 200 × 200 pixels. This process generated 107,305 images for the small buffer, and 105,441 images for the large buffer. These numbers differ as a small number of the large buffers did not fall entirely within the acquired aerial images. While all images were used to train the CAEs, only points with both a small and large buffer were included in the final feature extraction.

For each point and associated images, RTCs were assigned if they occurred within 50 m of each point. This process allowed for a single RTC point to be assigned to multiple road segments if it occurred somewhere in the middle, while also ensuring that no points are left unassigned to a road segment. Additionally, RTC data was aggregated for the years 2017–2020 so each image was assigned four years of RTCs to help account for some of the natural variance that can cause the number of RTCs in a particular location to fluctuate from year to year. We found that imagery, road network, and RTC data aligned closely enough to avoid necessitating further adjustments to the combined data. Samples of the combined data are provided in Supplementary Figure S1.

**Figure 2:**
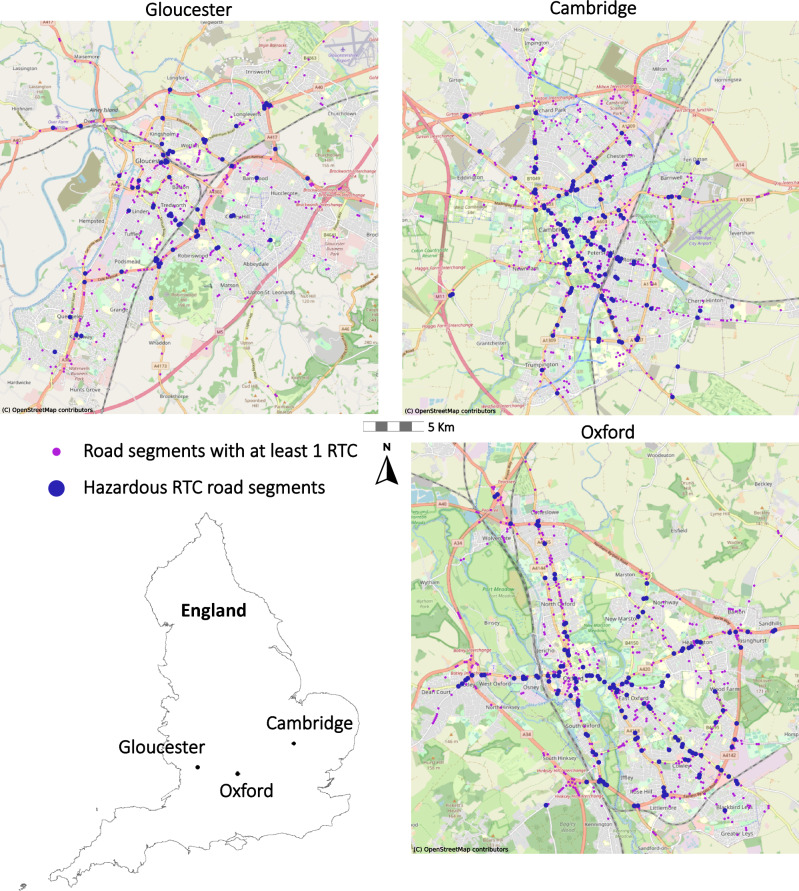
Locations of RTC and hazardous RTC road segments across the three UK towns. Maps were created in Python using software v3.9.15 (https://www.python.org/) and base maps from OpenStreetMap contributors (https://www.openstreetmap.org/).

Figure 2 maps out the location of each of the 1575 road segments which contained at least one RTC in each of the three cities under study. For final analyses, this study focuses only on 334 hazardous road segments, also shown in Fig. 2, which are defined here as points that contained at least three RTCs over the study period, similar to definitions used throughout Europe^48^.

It is worth noting that, due to the dense road networks in parts of the study cities, road segments that were sampled every 50 m along the street network could end up significantly closer than 50 m in straight line distance (especially when they occur around intersecting roads). While these segments contained relevant information to warrant inclusion in the model training and feature extraction steps, to avoid double counting similarly located segments in the clustering process, a further reduction of points was performed to ensure no segments remained within a 50 m buffer radius of any other point. A breakdown of the number of segments at each step in the analytic pipeline can be seen in Table 2.

**Table 2:** Data samples used throughout the analytic pipeline.

Locality	Image year selected for final analyses	# of segments used in training	# of segments used for feature extraction	# of segments after point reduction	# of hazardous road segments (> 3 RTCs)
Gloucester	2018	42,076	20,736	3,029	67
Cambridge	2020	32,022	15,676	2,776	129
Oxford	2019	33,207	17,142	3,154	138
Totals		107,305	53,554	8,959	334

### Feature extraction

Previous works have utilized CAEs to extract latent features from images^32,33^. By creating a convolutional network that can recreate an image, the feature space in the middle of the CAE forms a set of latent features that should contain a good representation of the data in a one-dimensional space. For this project, two identical CAEs were trained separately on the small and large image buffers respectively. The full model architecture can be found in Supplementary Figure S2. Each of the CAEs included 518,691 parameters split across an encoder and decoder culminating in a sigmoid activation. Both models were trained for 50 epochs using an Adam optimizer.

The input tensor to each of these CAEs is a 3 × 200 × 200 RGB image. Through the encoding portion of the model, whereby the latent structure of the images is learned, each image was compressed into a set of 4608 features. The size of this feature space was chosen to avoid over-compressing the data such that features could not be learned by the decoder algorithm, while also not creating so many features that would result in a sparse vector where features are hyper specialized to a small set of images.

While over 100,000 images were used to train each of the models, for the final feature extraction, only one timepoint from each locality was used to extract a final set of features. Timepoints were selected based on their temporal proximity to each other and their coverage of the study area which varied year over year. By including latent features from each of these two CAEs in the final clustering analysis, we expect that a set of built environment variables will be constructed that represent a holistic view of the area around each road segment, capturing both street-level and neighbourhood-level features.

### Dimension reduction and clustering

After extracting the latent features from the two CAEs, we next ran a PCA separately on each set of latent features to aid in dimension reduction. The PCA forms a representation of the latent feature space with a higher degree of interpretability than the features generated by the CAEs, while also concentrating a larger percentage of the variability from the image features into a smaller number of principal components^49^.

Agglomerative clustering was then performed on the combined sets of principal components to create a set of clusters from hazardous road segments. Importantly, the principal components computed from the latent image features likely contains common features that are shared by most images. To ensure a clustering analysis which could differentiate hazardous road segments, we only included a small subset of the principal components which displayed the highest correlation to at least one of the RTC variables. The number of highest correlated principal components selected varied by locale (Cambridge N = 946; Gloucester N = 73; Oxford N = 154). The final number of components used was determined iteratively by running the agglomerative clustering analysis on different sets of components and analysing the silhouette scores^50^ from 2 to 15 clusters to determine which sets of components with the highest correlation to RTCs also identified the most well-formed clusters.

Dendrograms in conjunction with the Calinski–Harabasz (CH) index were used to select the final number of clusters for each location. Dendrograms are a pictorial display of the hierarchical process of points being merged into successive clusters, with the lines connecting clusters representing the distance between each set of points^51^. The CH index on the other hand provides one evaluative measure of how well split clusters are using a variance ratio criterion^52^, this measure has been in used in previous research to evaluate the ideal number of clusters using agglomerative clustering^53^. Final clusters were chosen primarily due to their jump in linkage distance between merged groups, alongside visual inspection of cluster members, and their CH scores, where higher scores indicate a better internal split. This clustering analysis was done separately for Cambridge, Gloucester, and Oxford to allow some variance based on the unique combination of features that make up each city.

After clustering, descriptive correlational analysis using standard Pearson correlation coefficients and visual inspection of the clusters was performed to determine the utility of this process for augmenting road safety analysis. Clusters were examined to find potential outliers or interesting patterns on the various RTC variables. One cluster from each locale was then chosen to focus on and interrogate for potential usefulness. Shared aspects of each cluster’s road segments were identified which appear to be particularly associated with common RTC types within the cluster.

## Results and discussion

### Clustering hazardous road segments

**Figure 3:**
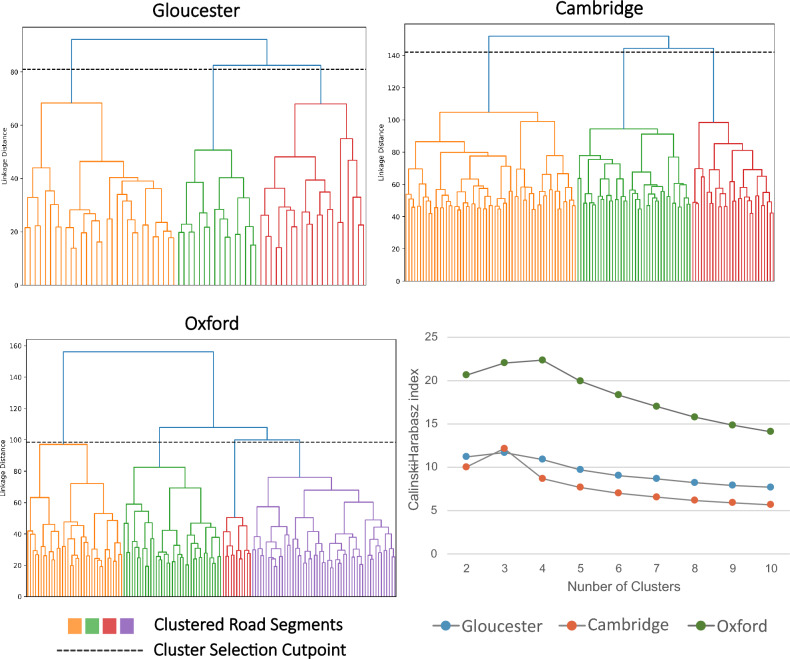
Cluster Selection. Dendrograms used to select the final number of clusters for the three UK towns alongside CH scores. Linkage distance and CH scores should not be compared across the different towns as it depends on the number of variables included in the clustering, which differed by locale. For CH scores, the optimal number of clusters are determined by the largest CH values.

Agglomerative clustering for Cambridge, Gloucester, and Oxford generated three separate dendrograms shown in Fig. 3. The dotted horizontal line in each plot signifies the cut point for choosing the number of clusters, whereby the number of intersecting vertical lines represents the number of final clusters. To assess which number of clusters best separated each of the datasets, the CH scores, also shown in Fig. 3 were used alongside the dendrograms to select the final clusters. For Cambridge and Gloucester, three clusters were determined to be most appropriate, while for Oxford four clusters of hazardous road segments were deemed most appropriate for this analysis. There is no one size fits all approach to selecting the number of clusters, exemplified by the fact that not each locale was determined to contain the same number of clusters.

### Descriptive analysis of clusters

**Figure 4:**
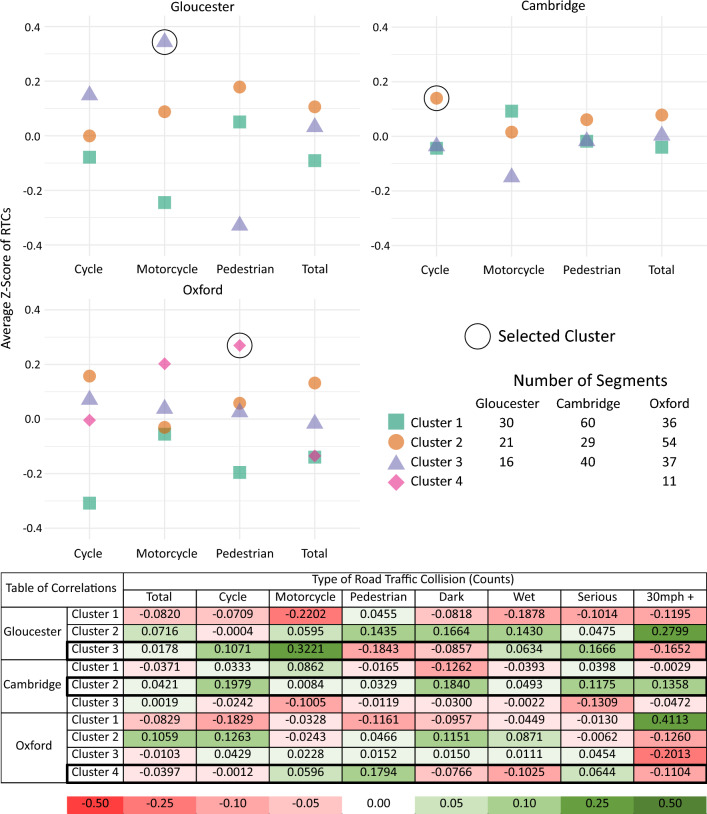
Descriptive Plots. The top dot plot shows selected RTC variables by cluster in each of the three UK towns. A Z-score of 0 denotes a mean level of that type of RTC for the specified locale, Z-scores should not be compared across locales. The bottom correlation table highlights relational patterns between membership in the clusters and the reported RTC data within each study site using standard Pearson correlation coefficients.

After identifying the final number of clusters in each location, descriptive analyses examined the various RTC variables to see if there were differences across clusters. Figure 4 shows selected RTC variables from each locale highlighting some of the most interesting RTC features. The goal of this descriptive analysis was to evaluate the utility of the clusters for identifying patterns within the RTC data. For this report, we selected one cluster of interest from each locale to examine in detail based on variations we observed among the different RTC variables. Imagery from the remaining clusters are shown in Supplementary Figure S3. Looking at the breakdown of RTCs by type within each cluster of Gloucester, Cluster 3 combines road segments with a higher average rate of RTCs involving motorcycles than either of the other two clusters, while also having the highest correlation to serious RTCs. In Cambridge, Cluster 2 stands out as having a high average number of RTCs involving cyclists, with small correlations to RTCs that occurred in the dark and at higher speeds above 30 mph. Alternatively, in Oxford RTCs involving pedestrians were observed at a higher rate in the smallest cluster, Cluster 4. These three clusters are examined qualitatively through visual inspection and mapping to explore the types of insights that can be gained from analysis which is augmented by this type of method.

### Visual interpretation of clusters

**Figure 5:**
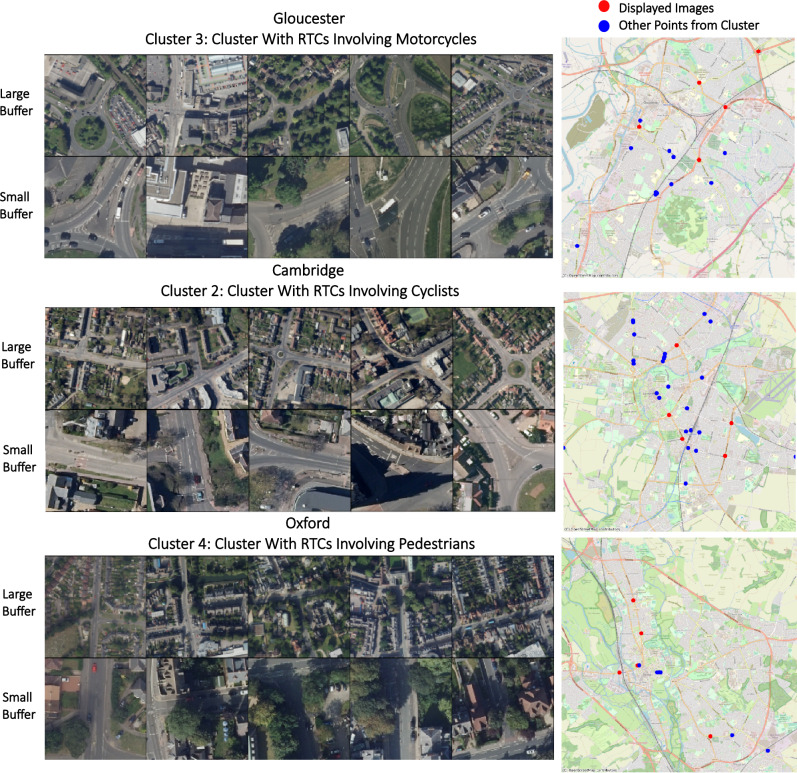
Visual interpretation of the selected cluster for each of the three UK towns. The maps on the right only show the location of hazardous road segments from the selected cluster, with the randomly chosen images highlighted in red. Maps were created in Python using software v3.9.15 (https://www.python.org/) and base maps from OpenStreetMap contributors (https://www.openstreetmap.org/). Imagery were sourced from the EDINA Aerial Digimap Service (https://digimap.edina.ac.uk/aerial).

Figure 5 shows a visual inspection of five randomly chosen road segments from the chosen clusters. Each road segment is shown with their two paired buffers. Policymakers from these cities will be best positioned to interpret the clusters and determine what interventions might be appropriate, however there are some high-level conclusions that can be made from a simple visual inspection. For instance, Cluster 2 from Cambridge contains road segments which all highlight long straight roadways with merging lanes. This cluster also has a high rate of serious RTCs, likely suggesting higher speeds are involved. The danger of these higher speeds could be further enhanced by the curved nature of these intersections. In line with their goals to create low-speed environments, Cambridgeshire has recently begun fast-tracking lower speed limits in selected zones throughout the county to combat this very issue^54^, highlighting one possible intervention which could be used to help reduce RTCs in this cluster.

In Gloucester, roundabouts appear in nearly all the images of the selected cluster, Cluster 3. Given the high rate of RTCs involving motorcycles within this cluster, it could be that motorcycles have particular difficulties with roundabouts in Gloucester, perhaps emphasizing the need for clarity in their right of way assignments. This echoes Gloucestershire’s recent Local Transport Plan which emphasized a need for interventions of education programmes aimed at motorcyclists^38^.

Looking at the chosen Oxford cluster, Cluster 4, these road segments contain the highest rate of RTCs involving pedestrians and appear to be in denser urban areas with T road segments. This could indicate places where pedestrians need to cross the street, but don’t currently have a straightforward means to do so. Uncovering why these sorts of junctions have been particularly problematic for pedestrians will feed into Oxford’s goal of better understanding collisions, while targeting this cluster will be essential for Oxford as creating more pedestrian-friendly roads is a key component of Oxfordshire’s Vision Zero plan to eliminate fatalities from RTCs by 2050^39^.

The right side of Fig. 5 shows the geographical distribution of these selected clusters. In each locale there is occasional grouping of points along thoroughfares and around large junctions. Some geographical clustering is expected given the use of aerial images, especially since the large buffers may overlap in places. Despite this, for each location hazardous road segments with similar built forms are identified across large distances in disparate parts of each city. This highlights the difficulties faced by policymakers in tackling an issue that spans across diverse communities.

### Harnessing the built environment

Initial inspection of the feature extraction process and the created clusters indicates that the methodology presented in this paper can identify meaningful clusters of hazardous road segments in three UK cities. In each location, a group of hazardous road segments was reviewed which share similar image-derived features. Because these road segments share common characteristics pulled solely from their built form, when it is found that these road segments also share similar challenges, such as a propensity for RTCs involving pedestrians, policymakers should have more confidence that these locations can be targeted by a comparable set of interventions. Looking at the whole set of hazardous road segments, there is not a strong reason for public officials to believe that just because similar RTC incidents frequently occur at multiple locations, that the cause of those RTCs and therefore planned interventions should also be similar in those locations. By utilizing aerial image features, this work suggests that the similarities found in the built environment in different parts of these cities can be leveraged to create better targeted interventions at reducing future RTCs.

### Data-augmented decision making

This methodology provides a straightforward pipeline for road safety experts to harness the power of ML through aerial imagery for the improved provision of public roads. Notably, by designing a pipeline that utilizes unsupervised ML and clustering techniques but is intentionally not a fully unsupervised process, experts can be kept in the analytic loop to leverage their necessary domain knowledge for enhanced qualitative analysis of hazardous road segments. Much research utilizing ML in road safety research is focused around using the newest methods to make better predictions about where RTCs will happen in the future or identifying where RTC hot spots are. However, simple descriptive analyses of governmental datasets can inform policymakers exactly where the most problematic road segments are. Much of the difficulty around the provision of increasingly complex public services is figuring out how to leverage expert knowledge to allocate a limited set of public funds in the most effective manner. As more cities set ambitious goals to eliminate deaths from RTCs, the methodology provided in this paper lays out one data augmented process whereby road safety experts are enabled to employ aerial imagery and technical tools, which may be underutilized within the public sector, to support the development of targeted local plans and road safety policies.

### Limitations

Although this paper presents some promising findings, there are some important limitations to consider. Most notably, the relatively simple visual feature extraction method used in this paper is likely insufficient to capture all the RTC-related features contained within aerial images given the complexity and scope of the potential feature space. More advanced ML architectures, such as the addition of attention mechanisms^11^, may be able to better extract RTC-related features. Moreover, aerial images only provide a top-down perspective of roads, so other types of imagery such as street-view images or 3D views may allow for additional features aligned with the road user’s perspective to be captured.

Additionally, cluster analysis itself has some limitations. Methods which are used to determine the ideal number of clusters, such as the silhouette score, are not always reliable, expert knowledge is often required to make meaningful interpretations of groupings, and results can be difficult to replicate, especially with alternative clustering algorithms that make different assumptions about what constitutes a cluster^55^. Furthermore, refinement will be needed in coordination with public sector collaborators to ensure that this and similar tools can be integrated effectively into current public sector decision-making streams.

## Conclusions and future research directions

This paper presented a pipeline to extract features from aerial images to allow for the clustering of hazardous road segments in three UK cities. The clusters examined in this paper were able to identify high level patterns among the RTC data and appeared well formed upon visual inspection, allowing an interpretable framework from which to assess the possible interventions that may be appropriate for reducing certain types of RTCs which are particularly prevalent in these groups. This research was designed to demonstrate how aerial imagery and ML methods can be leveraged to aid decisions that should reduce RTCs on public roads and improve governmental decision making. Building off this work, it is easy to see how similar methodologies could utilize aerial imagery to better understand other factors related to the built environment such as crime, energy use, pollution, wildlife management, and food access, to name but a few.

## Supplementary Information

Supplementary Information.

## Data Availability

The aerial imagery used in this study are available from the EDINA Aerial Digimap Service (https://digimap.edina.ac.uk/aerial) to members of subscribing higher and further education institutions in the UK. All other data sources are publicly available via OpenStreetMap (https://www.openstreetmap.org/) and the UK’s Department for Transport. The code used in this study can be found at: https://github.com/ai-for-public-services/sat-img-demonstrator.
