# Supplementary material for: Unsupervised feature extraction of aerial images for clustering and understanding hazardous road segments
Source: Sci Rep. 2023 Jul 5;13:10922. doi: 10.1038/s41598-023-38100-1 (PMC10322896; doi:10.1038/s41598-023-38100-1)

Supplementary Information

Table S1. Images and years used

Cambridge	Gloucester	Oxford
2016 (77 images)	2018 (100 images)	2016 (86 images)
2020 (77 images)	2021 (100 images)	2019 (100 images)
All images are 1km by 1km.		

Table S2. RTC Variable List and Frequencies

Variable	Cambridge	Gloucester	Oxford	Total
Total	1,423	788	1,316	3,527
Cycle	747	176	565	1,488
Pedestrian	178	150	189	517
Motorcycle	124	117	155	396
Serious	320	226	196	742
Dark	369	218	365	952
Wet	342	227	337	906
Time of Day				
Morning	330	185	309	824
Middle of Day	369	180	321	870
Afternoon/Evening	540	320	479	1,339
Night	184	103	207	494
Speed Limit				
20	248	29	566	843
30	962	532	323	2,017
40-50	94	161	131	386
60+	119	66	96	281
# of vehicles				
1	247	197	257	701
2	1,070	526	954	2,550
3	82	48	90	220
4+	24	17	15	56
# of casualties				
1	1,264	659	1,151	3,074
2	123	88	122	333
3	27	27	28	82
4+	9	14	15	38

Figure S1. Data Alignment. Maps were created in Python using software v3.9.15 (https://www.python.org/) and imagery were sourced from the EDINA Aerial Digimap Service (https://digimap.edina.ac.uk/aerial).

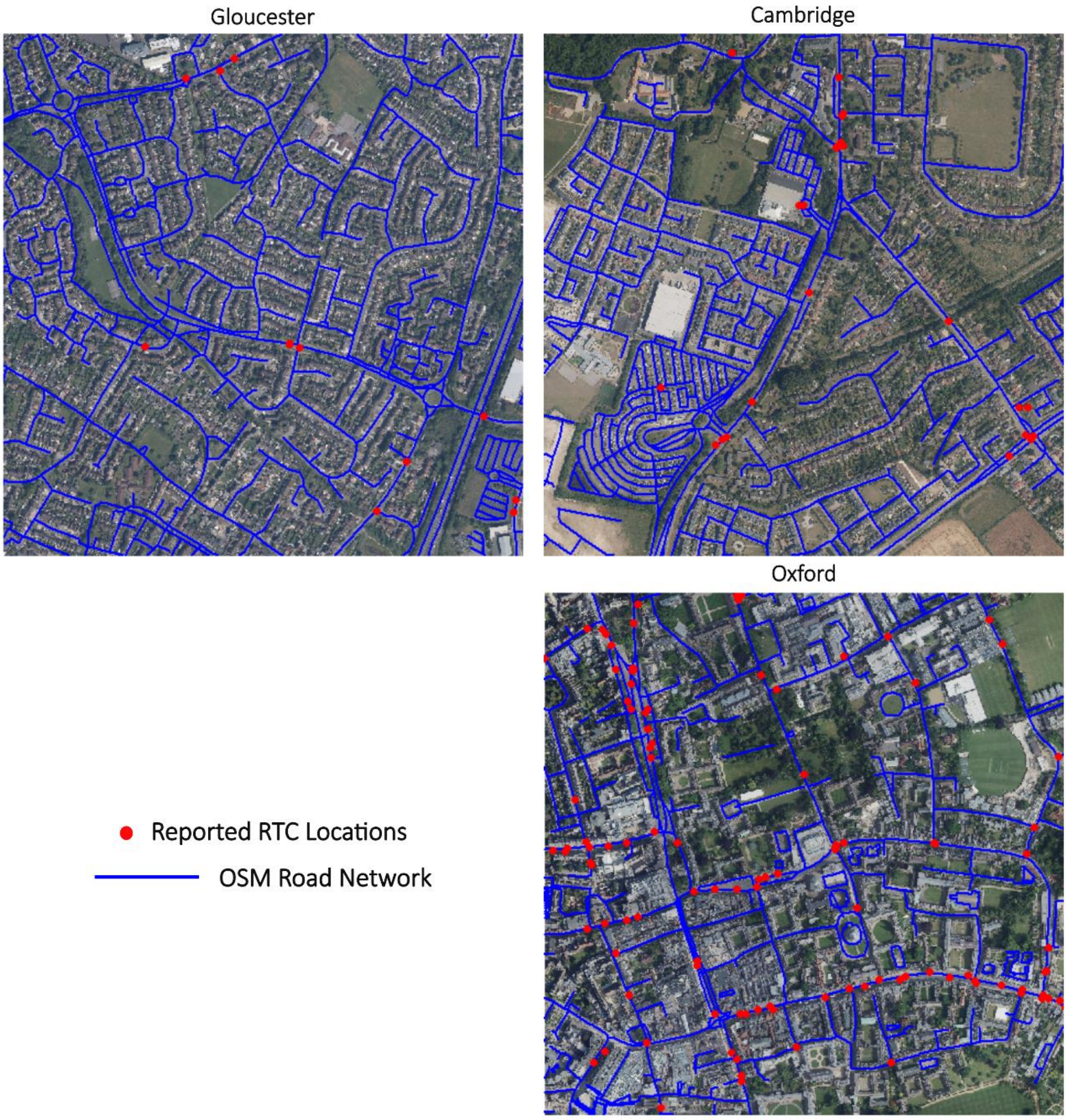

Figure S2. Model Architecture. Imagery were sourced from the EDINA Aerial Digimap Service (https://digimap.edina.ac.uk/aerial).

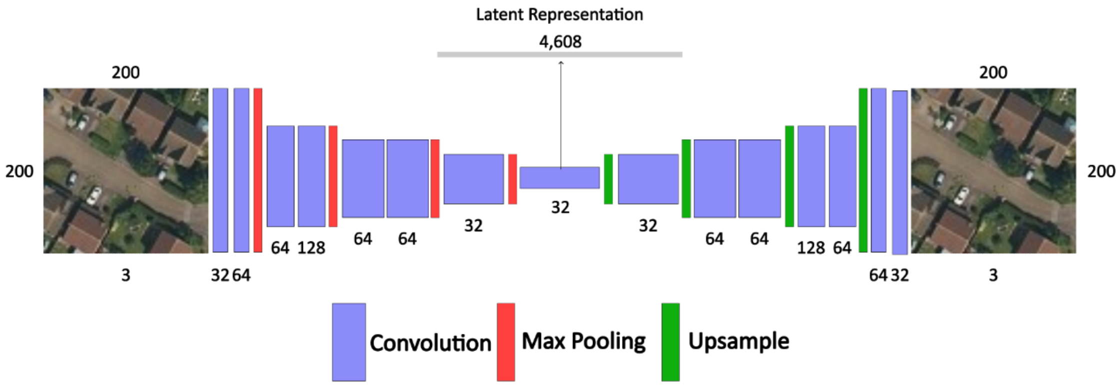

Figure S3. All Clusters

Five randomly selected images from the clusters of each study site are shown below at the 100m buffer. Details on the correlations between each cluster and different types of RTC incidents can be found in Figure 4 of the main text. Imagery were sourced from the EDINA Aerial Digimap Service (https://digimap.edina.ac.uk/aerial).

Gloucester Clusters

Cluster 1 Cluster 2 Cluster 3

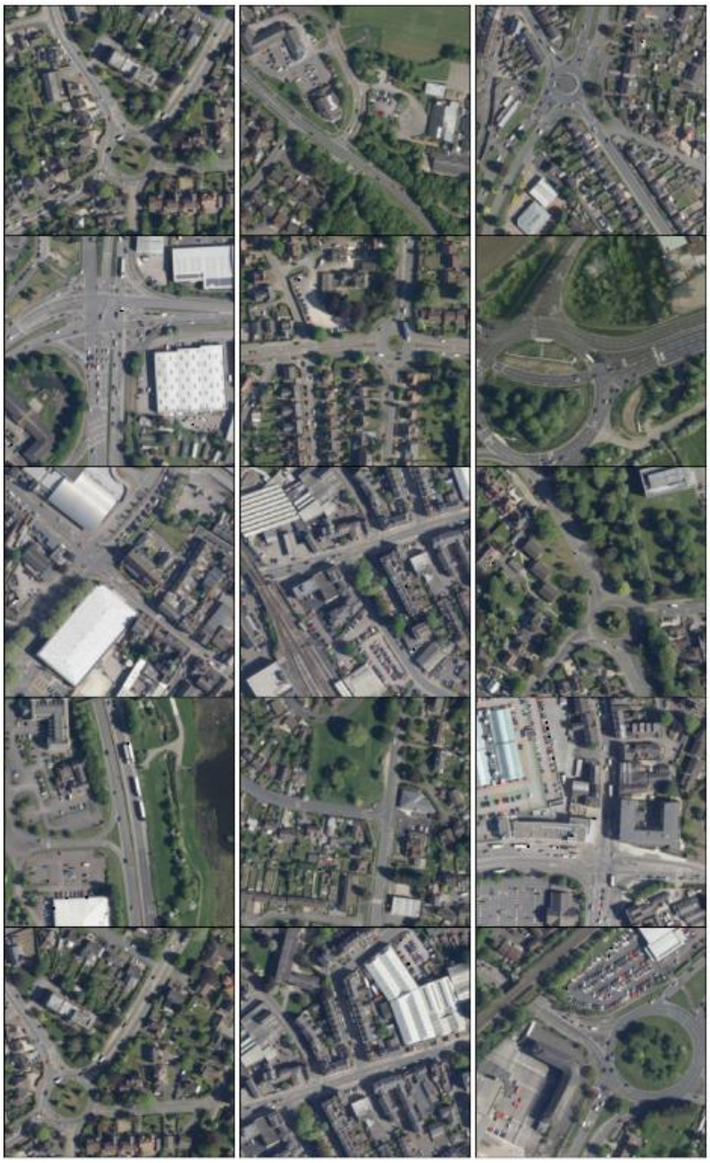

Cambridge Clusters

Cluster 1 Cluster 2 Cluster 3

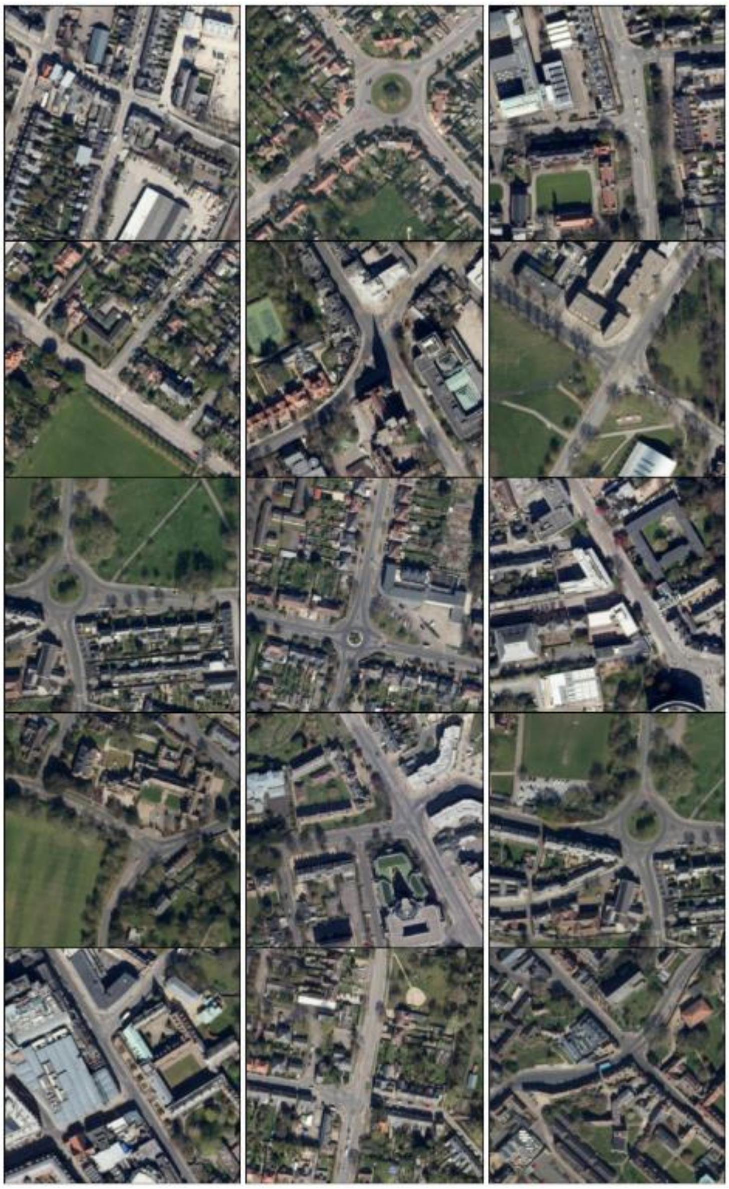

Oxford Clusters

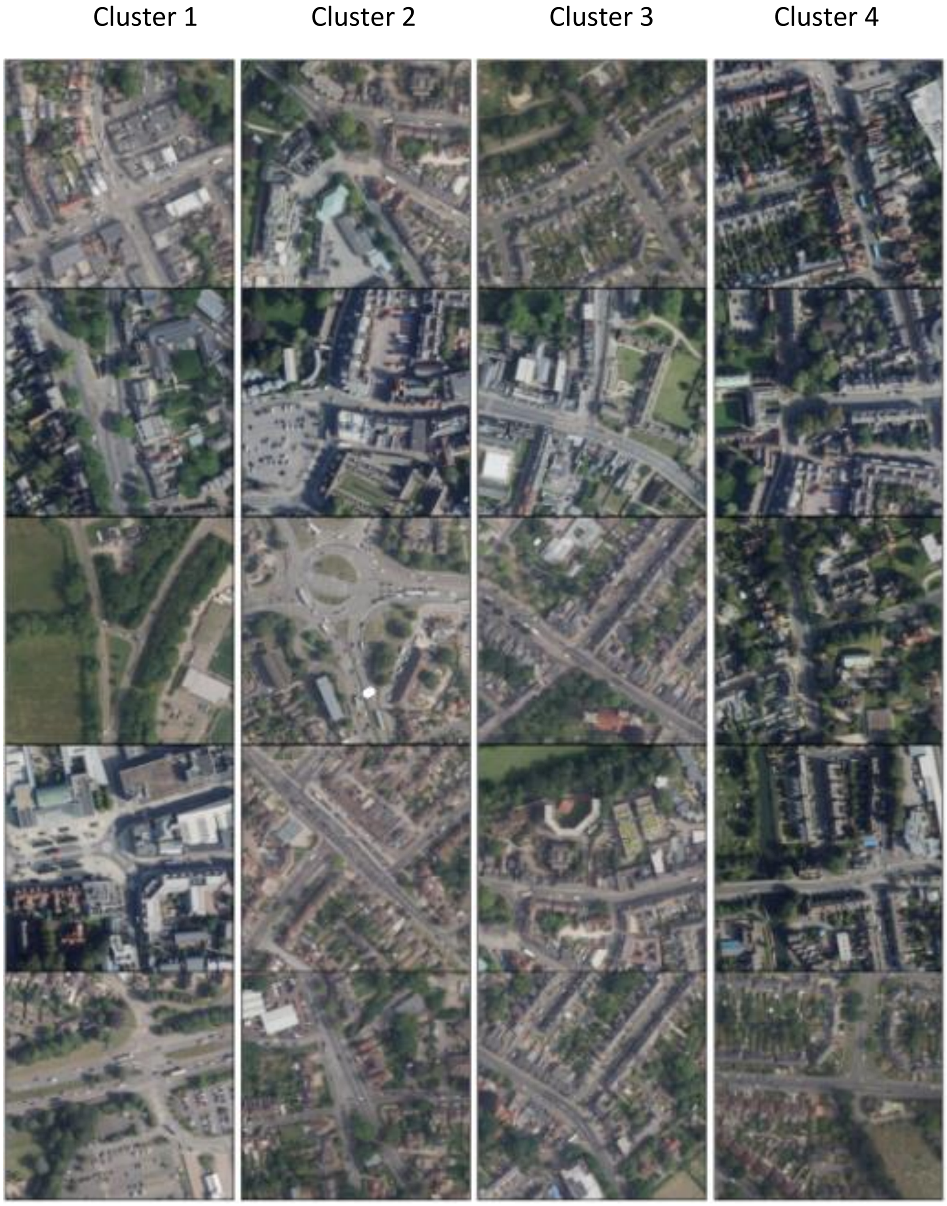

Supplement: Supplementary file 1 — Supplementary Information. [file 41598_2023_38100_MOESM1_ESM.pdf]
